# An Overview on Effects of Processing on the Nutritional Content and Bioactive Compounds in Seaweeds

**DOI:** 10.3390/foods10092168

**Published:** 2021-09-13

**Authors:** Ana Rodríguez-Bernaldo de Quirós, Julia López-Hernández

**Affiliations:** Department of Analytical Chemistry, Nutrition and Food Science, Faculty of Pharmacy, University of Santiago de Compostela, 15782 Santiago de Compostela, Spain; julia.lopez.hernandez@usc.es

**Keywords:** seaweeds, processing technologies, extraction technologies, bioactive compounds, nutritional value, seaweed-based food products

## Abstract

The effect of the different processing technologies and the extraction techniques on the bioactive compounds and nutritional value of seaweeds is reviewed in this study. This work presents and discusses the main seaweeds treatments such as drying, heating, and culinary treatments, and how they affect their nutritional value, the bioactive compounds, and antioxidant capacity. Some examples of traditional and green extraction technologies for extracting seaweeds bioactive components are also presented. The last trends and research on the development of seaweed-based food products is also covered in this review. The use of environmentally friendly extraction procedures, as well as the development of new healthy seaweed-based foods, is expected to grow in the near future.

## 1. Introduction

Nowadays, there are numerous studies concerning algae as a natural source of bioactive compounds with beneficial effects on health. Several therapeutic effects have been attributed to them, including anticancer, antiobesity, antidiabetic, antiviral activity, antioxidant, anticoagulant, anti-inflammatory, antihypertensive, reduce total and LDL cholesterol, and reduce the white adipose tissue weight, among others. Bioactive compounds are present in seaweeds comprise, polysaccharides and related compounds, polyphenols, phlorotannins, minerals, carotenoids, and so on [1,2,3,4]. According to their natural pigments, marine algae can be classified into three classes: green (Chlorophyceae), brown (Phaeophyceae), and red (Rhodophyceae) [5]. In Table 1, the chemical structure and properties of some examples of active compounds identified in algae are shown.

Some Asian countries (e.g., Japan, China, and Korea) have traditionally used algae in medicine and include them as one more element of their diet [6,7]. Seaweeds not only provide bioactive compounds but also have a significant nutritional value. Thus, dietary fiber, proteins, and polyunsaturated fatty acids form part of their composition [8,9].

In order to preserve food and guarantee the quality and safety and also increase the shelf-life of the products, different processing methods have been applied. In the case of seaweeds, they are commonly subjected to drying methods [10]. However, these procedures can have influence on the chemical composition of the product. There are many studies reported in the literature related to the effect of these procedures on the bioactive compounds as well as on the organoleptic properties [11,12,13,14]. Moreover, further culinary treatments may also lead to changes in the nutritional and chemical composition.

**Table 1:** Examples of the bioactive compounds identified in seaweeds.

Compound	Class	Chemical Structure	Properties	Reference
Fucoxanthin	Carotenoid	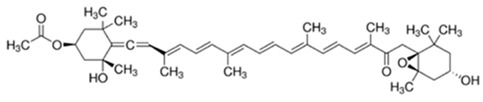	Antioxidant Reduce the white adipose tissue weight	[4]
α-Tocopherol	Vitamin	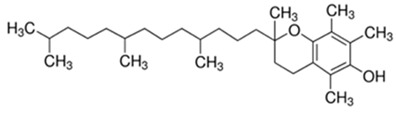	Antioxidant	[6]
Eicosapentaenoic acid	Fatty acid ω-3	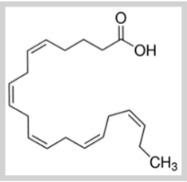	Reduce the risk of heart disease	[6]
Phloroglucinol	Polyphenols (phlorotannins)	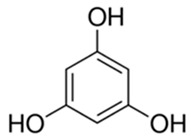	Antioxidant	[1]
Epicatechin gallate	Polyphenols	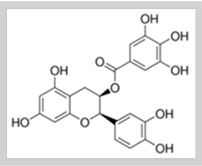	Antioxidant	[6]
Fucoidan	Sulphated polysaccharides	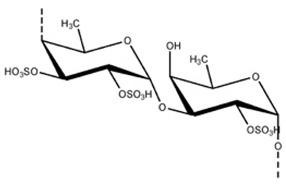	Antiviral activity	[6]
Phycocyanin	Phycobiliproteins	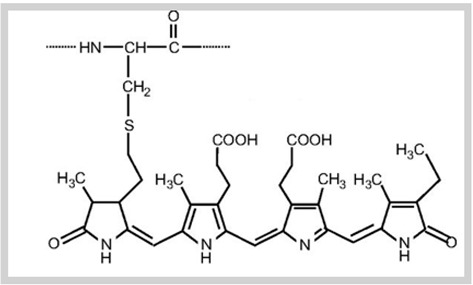	Antioxidant	[15]

This article is devoted to review the effects of the different processing technologies on the bioactive compounds and nutritional value of seaweeds. The manuscript is divided into three main sections. One is focused on the different processing technologies of seaweeds, including treatments for further commercialization (e.g., drying, canning, etc.) as well as culinary processes. It will discuss and analyze the effect of these treatments on bioactive compounds and nutritional value through the examples reported in the literature. In the second part it will comment on how the extraction technique (classical and new technologies) affects the bioactive compounds and antioxidant activity. The third section is dedicated to seaweed-based food products.

## 2. Effects of the Different Processing Technologies on Bioactive Compounds and Nutritional Value of Seaweeds

In the present section, the effect of common treatments to which the algae are subjected, including culinary treatments, heating and drying process, and so on, are described and commented on through studies reported in the literature.

In the study carried out by Pina et al. (2014), the effect of common treatments such as dried, hydrated, boiled, and steamed on the seaweeds’ components, including phycobiliproteins, carotenoids, volatile compounds, and antioxidant activity of samples of *Chondrus crispus*, were evaluated. The preparation of seaweeds was made according to the instructions specified in the packaging, namely, cooking in boiling water or cooking in steam for 20–30 min and 30 min, respectively. The rehydratation was achieved after 30 min in water. The phycobiliproteins were extracted with phosphate buffer and then determined by constant-wavelength synchronous spectrofluorimetry. For carotenoids, high-performance liquid chromatography with UV-Vis detector was employed, the analytical column was a Teknokroma Trace Extrasil ODS2 column (25 × 0.4 mm, 5 μm, particle size), and the mobile phase was a gradient consisted of methanol, acetonitrile, and hexane–dichloromethane (50:50, *v/v*). The antioxidant activity was determined by the DPPH (1,1-diphenyl-2-picrylhydrazyl) method. With respect to phycobiliproteins, the content of phycoerythrin in both dried and hydrated was similar (~530 mg/kg). On the contrary, an important decrease was observed in boiled and steamed samples (15.5–9.2 mg/kg), and for phycocyanin, dried and boiled samples presented comparable amounts (~150 mg/kg), hydrated samples presented higher levels (232.5 mg/kg), and it was not detected in steamed samples. The authors pointed out that the loss of phycoerythrin after culinary treatments could be attributed to the denaturation of the protein. The carotenoids analyzed in the study were β-carotene and lutein; β-carotene was only detected in boiled samples (0.88 mg/kg), whereas lutein was found in all types of samples except in dried samples. The values ranged between 0.64 and 2.80 mg/kg. In general, the authors observed an increase of carotenoids with the culinary treatments [15].

Amorim et al. (2012) evaluated the content of ascorbic acid, vitamin E, β-carotene, lutein, fucoxanthin, chlorophyll A, pheophytin a, total polyphenol, and antioxidant activity in fresh and cooked samples of *Laminaria* sp. (Kombu) and *Undaria pinnatifida* (wakame). Seaweeds were cooked by boiling with water. Ascorbic acid was determined by high-performance liquid chromatography with UV-Vis detection (HPLC-UV-VIs) after the extraction with aqueous 4.5% metaphosphoric acid; the column used was a Kinetex C18 (150 × 4.6 mm, 2.6 μm), Phenomenex^®^ and the mobile phase consisted of acetic acid: water (1:999) (*v/v*). For the carotenoids and vitamin E, the extracting solvent was a mixture composed by methanol–hexane–dichloromethane (50:25:25, *v/v/v*) and the stationary phase a Teknokroma Tracer Extrasil ODS2 column (250 × 4.6 mm, 5 μm particle size), carotenoids were detected at a wavelength of 450 nm and vitamin E was determined with fluorescence detection (λem 331 nm λex 288 nm). Total polyphenol content and antioxidant activity were determined by Folin–Ciocalteu’s and DPPH methods, respectively. Ascorbic acid was only detected in fresh wakame (118.7 mg/kg), and the content in vitamin E diminished in wakame cooked compared to fresh one (i.e., from 153.6 to 79.9 mg/kg). On the contrary, cooked kombu presented higher values with respect to fresh samples (24.1 vs. 9.4 mg/kg). In general, the cooked samples exhibited higher amounts of carotenoids and Pheophytin a (Wakame: fucoxanthin 1700 vs. 1119.1 mg/kg, lutein 112.6 vs. 8.4 mg/kg, β-carotene 898.7 vs. 316.1 mg/kg, Pheophytin a 10964.2 vs. 332.5 mg/kg in the case of Kombu samples: fucoxanthin 839.2 vs. 724.2 mg/kg, lutein was not detected in any samples analyzed and β-carotene 200.3 vs. 96.3 mg/kg, Pheophytin a 4441.3 vs. 184.5 mg/kg), however Chlorophyll A was only found in fresh samples (7261.1 mg/kg in wakame and 4182.3 mg/kg in kombu) [16].

In another study conducted by the same authors [17], the effects on the bioactive compounds, including vitamins C, E, fucoxanthin, lutein, β-carotene, Chlorophyll A, and phaeophytin a, and the antioxidant activity of dried edible seaweed stored under controlled conditions (darkness, room temperature) over time were evaluated. The extraction procedure of the carotenoids and liposoluble vitamins was performed by following the method reported by Ferraces-Casais et al. (2012) [18] with minor modifications, and the separation of compounds was achieved by using a reversed-stationary phase Tracer Extrasil ODS2 (25 × 0.4 cm, 5 μm particle size). Ascorbic acid was analyzed as described above. The antioxidant activity was determined using a DPPH-based method [19] and the total polyphenol content was determined according the Folin–Ciocalteu method. Dehydrated seaweeds *Himanthalia elongata*, *Laminaria* sp., *U. pinnatifida*, *Palmaria palmata*, and *Porphyra umbilicalis* were used in the study. The seaweeds were packed in polypropylene bags, stored, and then analyzed directly after processing and after 3, 6, 12, and 18 months. In general, red algae contained lower amounts of the bioactive compounds compared to the brown ones. At the time, 0 *H. elongata* presented the highest levels of vitamin C, E, polyphenols, and antioxidant activity. Ascorbic acid decreased significantly over time, which can be explained because of the oxidation process. In all seaweed tested it was only detected at time 0, except in *H. elongata*, where it was detected after 6 months of storage. Carotenoids, particularly β-carotene and lutein, decreased along the storage period. In both brown and red algae (*P. palmata*) chlorophyll A diminished, whereas Phaeophytin a increased in *Laminaria* sp. Overall, the antioxidant activity and the bioactive compounds decreased in dehydrated and packed samples compared with fresh samples, probably due to the oxidation process.

In another work, Amorim-Carrilho, et al. 2014 [20] evaluated the effects of the different culinary treatments on the vitamin C, vitamin E, fucoxanthin, chlorophyll A, pheophytin a, lutein, β-carotene, total polyphenol content, and antioxidant activity of *H. elongata*. The samples were analyzed dried, as they are in the package, boiled, steamed, and rehydrated (submerged 10 min in water). Analysis was performed according to the methods described above. Rehydrated seaweeds showed the highest values of all compounds except for β-carotene and pheophytin a, whose levels increased with boiled and steamed treatments. Boiled seaweeds presented higher antioxidant activity than dried samples (16,137.1 vs. 7392.4 μmol Trolox/kg dry matter). On the other hand, vitamin C was only found in dried seaweeds.

The effect of three drying methods comprising sun-drying, oven-drying, and freeze-drying on the nutritional properties of the seaweed *Sargassum hemiphyllum* was evaluated in a study carried out by Chan et al. (1997) [10]. Dietary fiber, protein, lipid, ash, as well as amino acids, fatty acids, minerals, and vitamin C were analyzed. The nutrients were determined as follows: amino acids were determined using an amino acid analyzer; fatty acids were determined as their methyl esters by gas chromatography-flame ionization detector (GC-FID) using a fused silica gel capillary column (SP-2560, 100 m × 0.25 mm i.d.); the analysis of the minerals was performed by inductively coupled plasma-atomic emission spectroscopy (ICP-AES) and the vitamin C was determined by the 2,4-dinitrophenylhydrazine method. The sun-drying method involves drying the sample under direct sunlight for 4 days. With the oven-drying method, the sample was dried in an air oven under the following conditions (60 °C, 15 h), and the freeze-drying consisted of freezing the sample at −70 °C in a freezer for 24 h, and after that it was dried in a freeze-drier for 5 days. Dietary fiber and ash were the main components of the seaweeds. On the other hand, the authors observed that in the case of protein and lipid there was not significant differences among the three treatments. The seaweeds dried by the sun-drying method presented the highest content in moisture and the lowest in ash. Regarding the amino acids content, the differences were not statistically significant among the samples dried with the different procedures; on the contrary, the amount of polyunsaturated fatty acids in freeze-drying samples was higher compared to sun- and oven-drying samples. In general, sun-dried samples presented the lowest levels of minerals, with the exception of the calcium. The levels of ascorbic acid in freeze-dried samples were significantly higher than that determined in sun- and oven-dried samples. This could be explained because the high temperatures reached during the treatments leads to a loss of the vitamin. The authors conclude that the freeze-drying treatment preserves the nutritional properties of the seaweed but is more expensive compared with the other treatments.

Jiménez-Escrig et al. (2001) [21] evaluated the antioxidant activity of different seaweed (*Fucus vesiculosus*, *Laminaria ochroleuca*, *U. pinnatifida*, *Ch. crispus*, *P. umbilicalis*, by three methods, namely, DPPH free radical-scavenging assay, FRAP assay, and in vitro copper-induced oxidation of human low-density lipoprotein assay. Fresh and processed (dried) Kombu and Nori samples were analyzed. The authors concluded that fresh seaweed presented higher activity compared to the processed samples and brown seaweeds presented higher values of antioxidant activity; among the species studied, *Fucus* showed the best results.

The effect of the rehydration conditions on the phytochemicals (total polyphenol content and total flavonoid content) and the antioxidant activity, which was determined by means of the DPPH radical scavenging test, of seaweed extracts of *H. elongata* was evaluated in a study reported by Cox et al. (2012a) [22]. The rehydration process was carried out at different temperatures (20, 40, 60, 80, and 100 °C). With respect to bioactive compounds, the total polyphenol content of the dried seaweed sample was 1.21 ± 0.02 g GAE/100 dry basis, and after the rehydration a significant loss within the first 10 min was observed at the temperatures studied; the total flavonoid content was reduced (88.3–93.2%) with increasing time and temperature. On the contrary, in the case of the antioxidant activity, the values increased after the treatment compared with the dried samples. In brief, the authors concluded that this treatment of rehydration results in a decrease of the bioactive compounds.

In a previous work, the authors studied the effect of heat processing on the color, bioactive compounds, and antioxidant capacity of three different species of edible Irish brown seaweeds, namely, *Laminaria saccharina*, *Laminaria digitata*, and *H. elongata*. Firstly, seaweeds were washed to remove impurities and stored under frozen temperature (−18 °C), then the samples were autoclaved at different temperatures (85, 95, 100, 110, and 121 °C) during 15 min. After that, they were crushed with liquid nitrogen and extracted with 60% methanol. Total phenolic content was determined by Folin-Ciocalteau’s method and expressed as mg gallic acid equivalents⁄g dry weight (dw). Total flavonoid content was determined by a colorimetric assay and was expressed as mg quercetin equivalents/g dry weight. Total condensed tannin content was also evaluated spectrometrically, using (+)-Catechin as the reference compound. Total sugar content was analyzed using the phenol–sulphuric acid method. With respect the antioxidant activity, this was evaluated by five different methodologies; DPPH radical scavenging assay, metal ion chelating ability assay, lipid peroxidation in a haemoglobin-induced linoleic acid system (LPO), hydrogen peroxide (H_2_O_2_) scavenging assay, and ferric reducing antioxidant power (FRAP) assay. The total phenolic content ranged between 35.8 ± 1.30 and 52.5 ± 1.65 mg gallic acid equivalents/g dry weight and increased with the temperature until 95 °C; higher temperatures resulted in a decrease of the TPC. Among the species studied, *H. elongata* showed particularly high values in both raw and treated samples. Regarding total flavonoid content, this also increased with the temperature until 85 °C. This increase was highest in *L. digitata*. On the other hand, *L. saccharina* presented the higher content of total condensed tannin in raw samples and after heating treatment. Additionally, total sugar content increased with the temperature. The authors concluded that this study demonstrates that the heating treatments leads to an increase in the antioxidant content [23].

In another work, the authors investigated the effect of different processing methods including drying pretreatment, boiling, steaming, and microwaving on the phytochemicals—total polyphenol content, radical-scavenging activity, total flavonoid content, total condensed tannin content, and antimicrobial activity—of the edible Irish seaweed *H. elongata*. The drying pretreatment was performed in a drier at a temperature of 25 °C for 12–24 h. The seaweeds were boiled in a water bath at 80–100 °C until a suitable texture was obtained. For the steaming process and microwaving, an atmospheric steam cooker and a conventional microwave oven were used, respectively [24]. Firstly, the samples were extracted with methanol 60% and then evaporated until dryness. Total polyphenol content was determined by the Folin–Ciocalteau method, radical-scavenging activity was carried out by DPPH, total flavonoid content and total condensed tannin content were determined according two methods reported in the literature, specifically Zhishen et al. (1999) [25] and Julkunen-Titto (1985) [26], respectively. The antimicrobial activity was tested against *Listeria monocytogenes, Salmonella abony*, *Enterococcus faecalis*, and *Pseudomonas aeruginosa*. With respect to the total polyphenol content, the boiling process leads to a loss around 80%, whereas with the steaming process a loss about 30% was observed when compared with the fresh seaweeds. On the other hand, the best radical-scavenging activity was provided by the seaweed dried for 12 h and then boiled at 100 °C for 25 min. The drying process has not an important effect on the total flavonoid content, however the boiling process leads to a significant decrease. Likewise, the total tannin contents are considerably reduced after the boiling process. The antimicrobial activity was influenced by the different processing treatments, and the highest antimicrobial activity was found in the fresh samples [24].

Sánchez-Machado et al. (2004a) [8] evaluated total lipid, protein, ash, and fatty acid contents in canned (*Saccorhiza polyschides* and *H. elongata*) and dried (*H. elongata*, *L. ochroleuca*, *U. pinnatifida*, *Palmaria* sp. and *Porphyra* sp.) edible seaweed. The drying conditions used were 45 °C for 24 h, then the samples were stored 3 days at room temperature and finally were packed in polypropylene bags. Canned samples were sterilized at 112 °C for 40 min, and these last samples were dried at 40 °C during 48 h prior to the analysis. Total lipid content was determined by using a mixture of chloroform–methanol (2:1 *v/v*) as extracting solvent. Fatty acids were analyzed as their methyl esters by GC-FID. The extraction and methylation of fatty acids was performed in one step using 5% methanolic HCl and toluene, and the separation was performed in a capillary column (30 mm × 0.32 mm, 0.25 μm) Supelco-Wax. Regarding the main fatty acids found in the seaweed (C16:0, C18:1ω9 and C20:4ω6), there was not statistically significant differences between dried and canned samples, however, regarding polyunsaturated fatty acid (PUFA) contents, there were statistically significant differences between two types of samples. Low levels of total lipid content were detected in all samples (<2%). The protein content ranged from 10.95 ± 0.27 (*H. elongata*) to 13.10 ± 0.12 (*S. polyschides*) for canned samples and from 5.46 ± 0.16 (*H. elongata*) to 24.11 ± 1.03 (*Porphyra* sp.) for dried samples, expressed as g/100 g dry weight.

In another work carried out by Norziah and Ching (2000) [27], the authors also determine the nutritional composition (total lipid, protein, fiber, ash, vitamin C, minerals β-carotene, free fatty acid, and amino acids contents) of a red seaweed *Gracilaria changii*. Because vitamin C and fatty acids are sensitive to light, temperature, and so on, samples were analyzed fresh, the rest of the nutrients were analyzed in dry samples. Vitamin C was determined by using a titrimetric method, and the fatty acid methyl esters (FAMEs) were analyzed by gas chromatography-flame ionization detector (GC-FID). An Omegawax 320 (30 mm × 0.32 mm, 0.25 μm film thickness) was used for the separation of FAMEs. Compared to the results reported by Sánchez-Machado et al. (2004a) [8], *G. changii* also presented important amounts of palmitic acid (C16:0) and oleic acid (C18:1ω9); in addition, eicosapentaenoic acid (C20:5ω3) and docosahexaenoic acid (C22:6ω3) were detected in significant concentrations. Total lipid content was slightly higher (3.3%) than that reported by Sánchez-Machado et al. (2004a) [8] for *S. polyschides*, *H. elongata*, *L. ochroleuca*, *U. pinnatifida*, *Palmaria* sp., and *Porphyra* sp. Regarding the other components, it is interesting to point out the high concentration of fiber found (24.7%).

In a more recent work, Maehre et al. (2016) [28] observed that heat treatment increases the bioavailability of proteins from seaweeds. To carry out the study, the authors employed an in vitro gastrointestinal digestion model. The seaweeds selected to carry out the study were *P. palmata* (red seaweed) and *A. esculenta* (brown seaweed). *P. palmata* augmented the amount of bioaccessible protein without deterioration of the amino acid profile after boiling; however, in *A. esculenta* the same behavior was not observed.

Within the work conducted by Kuda et al. (2005a) [29], fresh and dry products of different seaweeds, including brown algae (*Scytosiphon lomentaria* (kayamo-nori, dried product) *Papenfussiella kuromo* (kuromo, fresh-raw product), *Nemacystus decipiens* (mozuku, fresh-raw product), and red algae (*Porphyra* sp. (nori, dried product), were used to study the phenol contents and antioxidant properties of the ethanolic and aqueous extracts obtained from the algae. The parameters investigated were the following: suppression of hemoglobin-induced linoleic acid peroxidation, reducing power, ferrous ion chelating, 1,1-diphenyl-2-picrylhydrazyl (DPPH) radical scavenging, and scavenging of superoxide anion radical-generated non-enzymatic system.

To determine the total phenol contents a modification of the Folin–Ciocalteu method was employed, a photometry assay for the antioxidant activity in a hemoglobin-induced linoleic acid system, the 1,1-diphenyl-2-picrylhydrazyl (DPPH) radical scavenging test was performed using the method of Blois. The authors found that the highest values correspond to the aqueous solutions of kayamo-nori, mozuku, and nori, and lower values were obtained with the ethanolic extracts. Regarding the antioxidant activity, the aqueous extract of Bayamo-nori presented the highest value of reducing power. In the DPPH radical scavenging activity assay, the highest values were obtained in the aqueous extracts, particularly nori. In general, in the five tests carried out and for the four seaweeds studied, the aqueous extracts showed a stronger antioxidant activity compared with the ethanolic extracts. On the other hand, both dried (kayamo-nori and nori) and fresh products (kuromo and mozuku) presented antioxidant activity.

The same authors (Kuda et al. 2005b) [30] studied the antioxidant activity of dried kayamo-nori (brown algae *S. lomentaria*) by means of different tests, namely, suppression of hemoglobin-induced linoleic acid peroxidation, reducing power, ferrous ion chelating, 1,1-diphenyl-2-picrylhydrazyl (DPPH) radical-scavenging, and scavenging of a superoxide anion radical-generated non-enzymatic system. To conduct the experiments, aqueous and ethanolic extracts were used. The first one showed strong antioxidant activity compared with the alcoholic. Among the tests performed, the higher values were achieved with the linoleic acid peroxidation assay. The authors point out that the antioxidant activity could be reduced during the drying and storage process, but more assays are required.

Later, the authors (Kuda et al. 2006) [31] investigated the antioxidant properties of dried haba-nori *Petalonia binghamiae* (brown algae), a typical food consumed in Japan, by means of the assays indicated above. In the study, the authors also reported the effect of the heating treatment on the antioxidant activity. The results showed that the water extract presented significant antioxidant activity in the assays carried out.

With respect to the effect of heating, in order to evaluate the effect of the temperature, the procedure to obtain the aqueous and ethanolic extract was performed at three different temperatures (room temperature, 85 °C, and 121 °C) during 1 h. In general, for the aqueous extract at 121 °C, the phenolic compounds extracted were higher compared to those extracted at room temperature, and on the contrary for the ethanolic extracts, the temperature did not influence the extraction.

More recently, Linton Charles et al. (2020) [32] conducted a study to investigate the effect of four different drying techniques, specifically sun, oven, vacuum, and freeze-drying on the color and antioxidant capacity of seaweeds (*Kappaphycus alvarezii* and *Sargassum duplicatum*). The antioxidant capacity was tested by DPPH (1,1 diphenyl-2-picrylhydrazyl) scavenging assay, ABTS (2,2′-azino-bis (3-ethylbenzothiazoline-6-sulphonic acid), scavenging assay, and FRAP assay (ferric reducing antioxidant power), and the determination of total phenolic and flavonoid contents was also carried out. The results showed that all drying techniques affected the phenolic content and antioxidant capacity of the two species of algae, although the oven-dried seaweed extracts presented the highest phenolic contents and the highest antioxidant capacity. The color was preserved using oven, vacuum, and freeze-drying as drying techniques. The authors concluded that oven drying at a temperature of 50 °C was the technique of choice.

## 3. Effects of Extraction Techniques on the Bioactive Compounds of Seaweeds

Prior to the analysis, the bioactive compounds were extracted and isolated from the seaweeds. During the extraction procedure, seaweeds are subject to drastic conditions of time, temperature, pH, pressure, solvents, etc., which can affect the bioactive compounds. Traditional procedures such as liquid extraction with solvents as well as more advanced techniques including enzyme-assisted extraction, microwave-assisted extraction, ultrasound-assisted extraction, supercritical fluid extraction, and pressurized liquid extraction among others have been applied [33]. Due to growing environmental concerns, the use of green extraction technologies to extract bioactive compounds from different natural matrices has attracted the interest of scientists. Thus, this topic has been recently covered by several reviews published in the scientific literature [34,35,36]. Moreover, some other article reviews have been focused on specific techniques such as sub- and supercritical fluid extraction [37,38] and pressurized hot water extraction [39].

**Figure 1:**
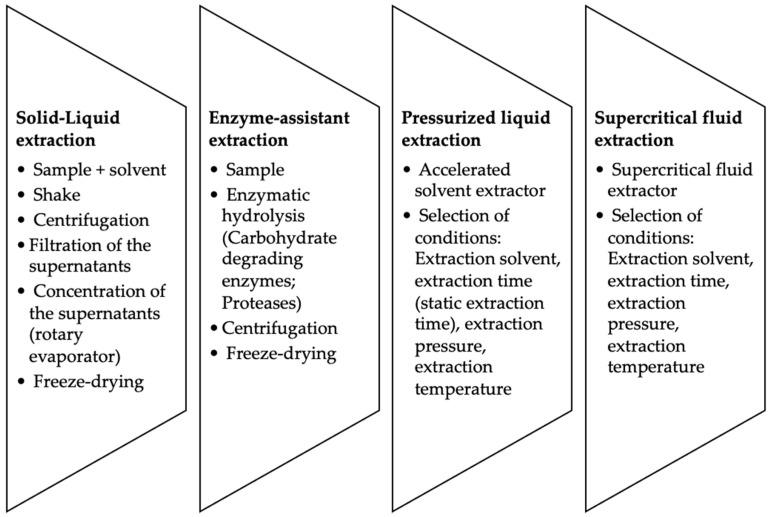
Scheme of protocols used for the extraction of bioactive compounds from seaweed.

In the present section, the effect of the extraction procedures on the compounds with biological activity are reviewed. Table 2 summarized the main extraction techniques used, and in Figure 1 a schematic representation of the main steps of the most common extraction procedures are shown.

A conventional solid–liquid extraction and pressurized liquid extraction were compared to extract polyphenols from the seaweeds *Ascophyllum nodosum*, *Pelvetia canaliculata*, *Fucus spiralis* and *Ulva intestinalis* collected in Ireland. Different solvents including water, ethanol–water (80:20 *v/v*), and acetone–water (80:20 *v/v*) were assayed and the pressurized liquid extraction was carried out using an accelerated solvent extractor. The total polyphenol content was determined by using the Folin–Ciocalteu reagent, and the antioxidant activity was evaluated by radical scavenging ability, ferric reducing antioxidant power, and ferrous ion chelating capability assay. The mixture of acetone–water (80:20 *v/v*) provided the best results for the total polyphenol content when pressurized liquid extraction was used. However, when solid–liquid extraction was applied, and for brown seaweeds, satisfactory results were obtained with water and ethanol–water (80:20 *v/v*). With respect to the antioxidant activity, the assays performed with solid–liquid extraction using the mixture of water and ethanol gave better results when compared with those made by employing the pressurized liquid extraction procedure for both DPPH and FRAP assays. The authors concluded that the high temperatures and pressures employed in the pressurized liquid extraction do not produce extracts with high antioxidant activity [40].

Ganesan et al. (2008) [41] studied the total polyphenol content and the antioxidant activity of the methanol extract, and five different solvent fractions obtained from three selected Indian red seaweeds, *Euchema kappaphycus*, *Gracilaria edulis*, and *Acanthophora spicifera*. The total polyphenol content was determined by using the Folin-Ciocalteau’s phenol reagent, the antioxidant activity was evaluated by means of the reducing power, the radical scavenging activity (DPPH), and the deoxyribose radical scavenging activity. The reducing power was estimated following the method proposed by Oyaizu (1986) [42], and the deoxyribose radical scavenging was determined according to Chung et al. (1997) [43] method. The solvent fractions studied were petroleum ether, ethyl acetate, dichloromethane, butanol, and aqueous. The petroleum ether fraction corresponding to *G. edulis* exhibited the highest polyphenol content 16.26 ± 1.40 expressed as mg gallic acid equivalents/g of seaweed extract, the lowest values were found in the butanol fractions of the three species analyzed. The methanol extract of *E. kappaphycus* presented the high radical scavenging activity, and regarding the solvent fractions, the petroleum ether showed the higher values in the three seaweeds. Regarding the deoxyribose radical scavenging activity in the dichloromethane, butanol, and aqueous fractions, the inhibition was higher. Therefore, the solvent fractions tested exhibited different antioxidant activity.

In a more recent work conducted by Sánchez-Caramago et al. (2016) [44], pressurized liquids and enzyme-assisted extraction were applied with the aim to develop an extraction procedure of phlorotannins from the algae *Sargassum muticum*. The total polyphenol content and the antioxidant activity in the extracts obtained were evaluated. The enzymatic treatment involves the use of proteases and carbohydrases. In addition, alkaline hydrolysis was performed to compare the results. Pressurized liquid extraction was carried out in an accelerated solvent extractor using ethanol and water, and to select the optimum conditions an experimental designed was used; the best conditions were 160 °C and 95% ethanol. Total phenols content and total phlorotannins content were determined by the Folin–Ciocalteu method and DMBA colorimetric assay, respectively. The antioxidant activity was evaluated by the Trolox equivalents antioxidant capacity assay (TEAC). The authors concluded that the combination of enzyme-assisted extraction with the pressurized liquids did not improve the extraction; therefore, the best solution was performing a single method based on pressurized liquids, and under the optimized conditions, the values of total phenols content, total phlorotannins and antioxidant activity were, respectively, 94.0 mg GAE g^−1^, 5.018 mg PGE g^−1^, and 1.275 mmol TE g^−1^.

The enzyme-assisted extraction was used to extract fucoxanthin and lipids containing polyunsaturated fatty acids (PUFAs) from a brown algae, *U. pinnatifida* or wakame. Seaweed samples were treated with alginate lyase and then the compounds, fucoxanthin, and lipids including fatty acids were extracted with dimethyl ether or ethanol. Various parameters such as type of solvent, amount of sample, and centrifugation were tested. Fucoxanthin was analyzed by high-performance liquid chromatography and diode array detector (HPLC-DAD) (λ 449 nm), a Waters Acquity UPLC BEH C18 (1.7 μm 2 × 150-mm) column, protected by a Waters Acquity UPLC BEH C18 1.7 μm VanGuard pre-column and a mobile phase consisted of 20 mM ammonium acetate and methanol were used; on the other hand fatty acids were determined as their methyl esters using gas chromatography with a flame ionization detector (FID) and a TraceGold TG-WaxMS (30 m × 0.25 mm i.d., 0.25 μm) capillary column. Best results were achieved with the enzyme pre-treatment followed by the extraction with dimethyl ether and ethanol as co-solvent, yields of fucoxathin of 94% and lipids of 94% were obtained, however when ethanol extraction was used, lower values were achieved by fucoxanthin (86%) and lipids (73%). High concentrations of ω-3 and ω-6 polyunsaturated fatty acids were also found [45].

In another study, the enzyme-enhanced extraction method was used for antioxidant ingredients from red algae *P. palmate*. The effect of protease and carbohydrase treatments was examined. The total phenol content and the antioxidant activity was evaluated by the 1,1-diphenyl-2-picrylhydrazyl (DPPH) radical scavenging activity, oxygen radical absorbance capacity (ORAC), and ferrous ion-chelating ability assays. Regarding the total phenol content, the groups treated with protease presented higher values, and in general the hydrolysates treated with carbohydrases were lower. With respect to the DPPH radical scavenging activity, the hydrolysates treated with protease were higher than that of water extract, with the exception of Alcalase extract. The authors concluded that the treatment with protease improved the extraction of the bioactive compounds [46].

Rodrigues et al. (2015) [47] applied the enzyme-assisted and ultrasound-assisted extraction to characterize the proximate composition (total nitrogen, total sugars content, total phenolic content) and to determine the antioxidant activity (total antioxidant capacity, the DPPH free radical scavenging activity, hydroxyl radical scavenging activity, and superoxide radical scavenging activity) in the seaweeds *S. muticum*, *Osmundea pinnatifida*, and *Codium tomentosum*. In addition, the determination of prebiotic potential of seaweed extracts was carried out. The total nitrogen content was determined by the Kjeldahl method, total sugars content was performed in accordance with the phenol−H_2_SO_4_ method [48] and the total phenol contents by the Folin–Ciocalteu method using catechol as the standard. For the enzyme-assisted extraction, different enzymes were checked, that is, carbohydrate-degrading enzymes including Viscozyme L and Cellulase and the proteases, alcalase, and flavourzyme. Additionally to the enzyme-assisted and ultrasound-assisted extraction, hot water extraction was carried out. The authors observed differences among the extraction procedures, the enzyme-assisted extraction seems to be the most effective method; the highest extraction yields were obtained when using both enzymes, carbohydrases, and proteases. On the other hand, with ultrasound-assisted extraction, the extraction was not higher than that obtained with hot water, except for *C. tomentosum*. In this case, a slightly higher extraction was achieved. In general, there were not differences between ultrasound-assisted extraction and hot water extraction in what concerns to total nitrogen content and total sugar content. *O. pinnatifida* was the seaweed with lower values of total antioxidant capacity and DPPH free radical scavenging activity and with higher values in the superoxide radical scavenging activity test. This algae also presented a lower prebiotic potential.

Blamo et al. (2020) [49] proposed ultrasound-assisted extraction (50 °C, 60 min, 30% ethanol) as an useful method to maximize the phenolic compounds and antioxidant activity obtained from the red algae *Laurencia intermedia*. Once more, the same authors employed this technique to extract phenolics from Vietnamese brown seaweed *Padina australis*, the optimal conditions were 60 °C, 60 min, and 60% (*v/v*) aqueous ethanol as solvent [50].

In the study reported by Siriwardhana et al. (2008) [51], total polyphenol contents and antioxidant activity were determined in the seaweed *Hizikia fusiformis.* In the extraction process several parameters were evaluated, pH, heat, and enzymatic hydrolysis. The pH varied between 2 and 12, the temperatures tested were 25, 50, 75, and 100 °C, and the protease alcalase and the carbohydrase Ultraflo were used. The total polyphenol contents were determined with the Folin−Ciocalteu reagent, and the antioxidant activity was evaluated by means of 1-diphenyl-2-picrylhydrazyl radical scavenging assay and hydrogen peroxide scavenging assay. The results indicated that higher pH leads to better extraction efficacy, therefore the optimum pH was 12. Regarding the temperature in the case of the 1-diphenyl-2-picrylhydrazyl radical scavenging, it was observed that higher temperatures resulted in an increase of the DPPH radical scavenging activity, however this behavior was not observed in hydrogen peroxide scavenging assay, and 100 °C was selected as the optimum temperature. On the other hand, the best proportion of the enzymes used was 2:3 and 5:0 of alcalase and ultraflo for the total polyphenol contents, 2:3 and 3:2 for the DPPH radical scavenging activity, and 2:3 for hydrogen peroxide scavenging activity.

Microwave-assisted enzymatic extraction was used by Charoensiddhi et al. (2015) [52] as an interesting method to extract phlorotannins and antioxidant compounds from a brown seaweed, *Ecklonia radiate*, recollected in South Australia. In developing the method several enzymes were tested, including the carbohydrases, viscozyme, celluclast, and ultraflo, and the proteases, alcalase, neutrase, and flavourzyme. On the other hand, the extraction was performed with microwave heating, using acid–base and water extractions as reference. The antioxidant activity was determined by ferric reducing ability of plasma (FRAP) and the oxygen radical absorbance capacity (ORAC) tests and the total phlorotannins were determined using the Folin-Ciocalteu reagent and phloroglucinol as standard. The treatment with the carbohydrases viscozyme, celluclast, and with a mixture of both enzymes with and without the microwave heating leads to a higher extraction efficiency of total phlorotannins compared to the conventional method with water. On the contrary, the use of the protease alcalase, neutrase, and flavourzyme or the carbohydrase ultraflo usually resulted in lower extractions. The acid extraction also gave suitable results. In the FRAP assay, the best results were achieved when using viscozyme, celluclast, or a mixture of them with and without microwave heating. Analogous results were obtained with the ORAC assay, although in this case the treatment with celluclast and acidic extraction did not enhance the ORAC values. In addition, the use of the protease alcalase together with the microwave heating exhibited the higher ORAC value.

In the study reported by Anäelle et al. (2013) [53], different and novel extraction methods such as centrifugal partition extraction, supercritical fluid extraction and pressurized liquid extraction, and the conventional solid–liquid extraction were investigated in terms of the extraction efficiency of total phenol contents, DPPH radical scavenging assay, and the β-carotene bleaching method from the brown seaweed *S. muticum*. Ethyl acetate: distillated water (50:50 *v/v*) was used as extracting solvent in the centrifugal partition extraction method. Supercritical fluid extraction was performed in a supercritical fluid extractor under the following conditions: CO_2_ modified with 12% ethanol at a pressure of 15.2 Mpa a temperature of 60 °C and an extraction time of 90 min. Pressurized liquid extraction was performed using ethanol 25% and 75% as extracting solvent. The methods assayed have different effects on the bioactive compounds. In the case of total phenol contents, the ethyl acetate–distillated water provided high results with both traditional and novel or alternative methods whereas the lowest values were obtained with CO_2_–ethanol and with the traditional method using hexane–ethanol. In DPPH radical scavenging assay, best results were achieved with the conventional methods and the highest values of antioxidant activity determined by the β-carotene bleaching method corresponded to the hexane–ethanol and the ethyl acetate extracts obtained with traditional and alternative methods, respectively.

The effect of different parameters, including the acid concentration (HCl) and the time and the temperature on the yield of fucose-containing sulfated polysaccharides obtained from the brown algae *Sargassum* sp., was studied [54]. One and two-steps extraction procedures were assayed, and several conditions such as 0.1 M HCl and 0.2 M HCl, reaction time (1 h, 3 h, 5 h), and temperature (30, 60, and 90 °C) were tested. The results indicated that the highest polysaccharide yield was obtained with the single step procedure under the following conditions: 0.03 M HCl, 90 °C, 4 h. On the contrary, the lowest values were found at 30 °C or 60 °C with 0.2 M HCl.

Lately, ionic liquid-assisted subcritical water extraction (SWE + IL) was investigated as a novel method to obtain phenolic compounds from *Saccharina japonica*; in the study, solid–liquid extraction (SLE) was also carried out as reference method. The results revealed that SWE + IL and SWE provided higher extraction of phenolic compounds compared to conventional SLE [55]. Another new extraction technique, namely autohydrolysis with compressed hot water, has been successfully applied to extract bioactive compounds from dehydrated *L. ochroleuca* [56].

**Table 2:** Overview of the main extraction methodologies for bioactive compounds of seaweeds.

Bioactive Compound	Extraction Technique	Seaweed	Results of the Study	Reference
**Polyphenol**	Solid–liquid extraction (SLE) Pressurised liquid extraction (PLE)	*A. nodosum* *P. canaliculata* *F. spiralis* *U. intestinalis*	Generally, PLE provided lower values of Total Polyphenol Content (TPC) compared with SLE, which could be due to the higher temperatures employed in PLE	[40]
**Polyphenol**	Pressurized liquids Enzyme-assisted extraction (EAE)	*S. muticum*	In terms of TPC the use of EAE in combination with PLE did not provide better results compared to the use of PLE alone	[44]
**Fucoxanthin and lipids**	Enzyme-assisted extraction	*U. pinnatifida*	The enzyme pre-treatment followed by the extraction with dimethyl ether and ethanol as co-solvent leads to better results compared to conventional extraction with ethanol	[45]
**Phlorotannins**	Microwave-assisted enzymatic extraction	*E. radiate*	The microwave-assisted enzymatic extraction provided better results in comparison with the conventional acidic extraction	[52]
**Polyphenol**	Centrifugal partition extraction (CPE) Supercritical fluid extraction (SFE) Pressurized liquid extraction Solid–liquid extraction	*S.muticum*	The new methods particularly, CPE and PLE using an EtOH:water mixture 75:25 (*v/v*) provided better results than the conventional solid–liquid extraction. The exception was SFE that gave poor results	[53]
**Polyphenol**	Subcritical water extraction (SWE) Ionic liquid-assisted Subcritical water extraction (IL + SWE) Solid–liquid extraction	*S. japonica*	SWE + IL and SWE provide better extraction efficiency than SLE	[55]
**Polyphenol**	Ultrasound-assisted extraction	*P. australis*	Several extraction variables were studied. The results showed that the extraction temperature, solvent concentration and sample-to-solvent ratio significantly affected the TPC, however the extraction time had not effect on TPC	[50]
**Phlorotannins**	Autohydrolysis with compressed hot water	*L. ochroleuca*	Autohydrolysis with compressed hot water seems to be an innovative and suitable extraction technique for bioactive compounds	[56]

## 4. Seaweed-Based Food Products

It is well-know that seaweeds are a natural source of bioactive compounds, such as polyphenols, carotenoids, fatty acids, certain types of vitamins, among others. In past years, different food products enriched with algae have been elaborated with the aim to obtain healthy products. Some examples reported in the literature are here described and commented on.

In the past years, in a work reported by Mišurcová et al. (2014) [57], the amino acid profile of several dried seaweed-based products obtained from the micro-algae, *Spirulina pacifica*, *Spirulina platensis*, *Chlorella pyrenoidosa*, and from the seaweed, *P. palmata, Porphyra tenera, Eisenia bicyclis, H. fusiformes*, *Laminaria japonica*, and *U. pinnatifida*, were evaluated. The amino acids were determined by ion-exchange chromatography, the method involved a derivatization step post-column using ninhydrin. Detection was performed at a wavelength of 440 nm for proline and 570 nm for the rest of the amino acids. As expected, different amino acid contents were obtained from the different species of the micro- and macro-algae and, as well, among the products prepared from the same seaweed. The contents of amino acids varied between 22.8–42.3 (g·16 g^−1^ N) and 31.0–66.5 (g·16 g^−1^ N) for essential and non-essential amino acids, respectively. The highest content in amino acids were found in *Spirulina Pacifica* and in a product made with *Eisenia bicyclis*. Moreover, *S. pacifica* presented the highest amount of essential amino acid and *E. bicyclis* showed the highest quantity of nonessential amino acids. Arame, a product obtained from *E. bicyclis*, exhibited the high content in sulfur amino acids. In general, the samples studied presented significant contents of amino acids.

The nutritional composition that is, minerals, trace elements, amino acids, fatty acids, and the phycobiliproteins C-phycocyanine and allophycocyanine in several products based on *Spirulina* and with different origins (Cuba, Italy, Mexico, and USA) were investigated. Minerals and trace elements were analyzed by Instrumental Neutron Activation Analysis and Inductively Coupled Argon Plasma-Atomic Emission Spectroscopy; amino acids were determined as their phenyltiocarbamil derivatives by reversed phase liquid chromatography with UV detector at 254 nm and using a Supelcosil LC 18-DB (Supelco, Bellefonte, PA, USA), (25 × 0.46 cm i.d., 5 μm) as a stationary phase. Fatty acids were determined by gas chromatography and flame ionization detector, the separation was performed on a SP-2380 fused silica capillary column, (30 m × 0.25 mm i.d.) and the analysis of phycobiliproteins was carried out by HPLC with UV detector. The values of minerals found, were generally similar to those reported in the literature for products based on *Spirulina*. On the other hand, *Spirulina* and the products prepared from this micro-algae presented significant amounts of phycobiliproteins. The sample from Cuba presented a high value of N-protein and also for the amino acids except for histidine, while the sample from USA presented the lowest values. However, the fatty acid profile was similar in all products evaluated [58].

In the study conducted by Prabhasankar et al. (2009) [59], the seaweed *U. pinnatifida* (wakame) was incorporated as an ingredient to elaborate pasta. The total phenol content, antioxidant activity, fatty acids, fucoxanthin and fucosterol were determined in the pasta enriched with the seaweed. Additionally, the sensory properties were studied. The antioxidant activity was evaluated by 1,1-Diphenyl-2-picrylhydrazyl (DPPH) and superoxide radical scavenging activities. Fucosterol and fucoxanthin were analyzed by HPLC with photo-diode array detector (λ 450 nm for fucoxanthin and λ210 nm for fucosterol) after an extraction with a mixture of chloroform and methanol (2:1; *v/v*). The column employed was a C30 column (Develosil C30 UG-5, 250 × 8.0 mm i.d., 5.0 μm particle size, Nomura Chem. Co., Aichi, Japan) and the mobile phase consisted of methanol and acetonitrile (70:30, *v/v*). The fatty acids were determined as their methyl esters by gas chromatography with a flame ionization detector and using a capillary column (Omegawax 320, 30 × 0.32 mm i.d., Supelco, Bellefonte, PA, USA). The enriched pasta with wakame increased the levels of proteins and fats compared to the control, and additionally presented fucoxanthin and fucosterol typical compounds of the seaweed. It is interesting to point out that the subsequent treatments of cooking do not affect these bioactive compounds. The total phenol contents were higher in the pasta containing seaweeds than the control pasta and the phenol contents augmented as the amount of algae incorporated increases. In DPPH assays, the highest values correspond to uncooked pasta with the wakame. Additionally, the enriched pasta exhibited high values of superoxide scavenging activity. The wakame contributed to increase the levels of fatty acids ω-3 in pasta.

Nagai and Yukimoto (2003) [60] reported the antioxidant properties of four beverages made from seaweeds. For that purpose, *U. pinnatifida, Ecklonia cava, H. fusifome*, and sea lettuce, *Ulva pertusa*, were used. The antioxidant properties were evaluated by means of total phenolic compounds, DPPH radical test, and superoxide-scavenging activity. The beverage type tea made with *E. cava* presented the highest amount of total phenols whereas the drink made with *U. pertusa* presented the lowest. Similar results were obtained in the superoxide-scavenging activity and in DPPH radical scavenging activity of beverages. According to the results obtained in the study, *E. cava*, also known as sea trumpet, presented a strong antioxidant activity.

Jayakody et al. (2021) [61] reported the development of a seaweed snack using *Ulva fasciata.* In order to enhance the flavor of the new product, ginger oleoresin was added after the drying process. The snack presented high mineral, protein, and fiber content.

Others interesting applications, such as the use of seaweeds or their bioactive compounds to elaborate meat products or the development of seaweed-derived bioactive compounds for use as prebiotics, have been recently reviewed [62,63].

## 5. Conclusions and New Trends

In summary, seaweeds are subjected to different processing treatments, mainly drying methods, in order to preserve them, ensure their quality and safety, and facilitate its commercialization. As it is well illustrated in this review, these procedures, together with the culinary processes to which the algae are subjected for their subsequent consumption, can affect the bioactive compounds and the antioxidant activity.

Regarding the extraction of the bioactive compounds, traditional methods usually involve the use of organic solvents or heat, which can negatively affect the active compounds extraction. It is expected that green technologies would be widely used in order to avoid the limitations of the conventional methods.

On the other hand, a clear trend in the near future is foods enriched with functional ingredients. In this sense, seaweeds are an excellent source of bioactive compounds.
